# Substitution of natural sensory input by artificial neurostimulation of an amputated trigeminal nerve does not prevent the degeneration of basal forebrain cholinergic circuits projecting to the somatosensory cortex

**DOI:** 10.3389/fncel.2014.00385

**Published:** 2014-11-14

**Authors:** Celia Herrera-Rincon, Fivos Panetsos

**Affiliations:** ^1^Neurocomputing and Neurorobotics Research Group, Universidad Complutense de MadridMadrid, Spain; ^2^Biomathematics Department, Faculty of Biology and Faculty of Optics, Universidad Complutense de MadridMadrid, Spain; ^3^Instituto de Investigación Sanitaria del Hospital Clínico San CarlosMadrid, Spain; ^4^Department of Industrial Engineering and Management Systems, University of Central FloridaOrlando, FL, USA

**Keywords:** magnocellular basal nucleus, acetylcholine, plasticity, barrel cortex, BMI, auditory cortex, visual cortex, basal forebrain

## Abstract

Peripheral deafferentation downregulates acetylcholine (ACh) synthesis in sensory cortices. However, the responsible neural circuits and processes are not known. We irreversibly transected the rat infraorbital nerve and implanted neuroprosthetic microdevices for proximal stump stimulation, and assessed cytochrome-oxidase and choline- acetyl-transferase (ChAT) in somatosensory, auditory and visual cortices; estimated the number and density of ACh-neurons in the magnocellular basal nucleus (MBN); and localized down-regulated ACh-neurons in basal forebrain using retrograde labeling from deafferented cortices. Here we show that nerve transection, causes down regulation of MBN cholinergic neurons. Stimulation of the cut nerve reverses the metabolic decline but does not affect the decrease in cholinergic fibers in cortex or cholinergic neurons in basal forebrain. Artifical stimulation of the nerve also has no affect of ACh-innervation of other cortices. Cortical ChAT depletion is due to loss of corticopetal MBN ChAT-expressing neurons. MBN ChAT downregulation is not due to a decrease of afferent activity or to a failure of trophic support. Basalocortical ACh circuits are sensory specific, ACh is provided to each sensory cortex “on demand” by dedicated circuits. Our data support the existence of a modality-specific cortex-MBN-cortex circuit for cognitive information processing.

## Introduction

As early as the 1970s it has been known that sensory deafferentation provokes changes in the somatotopic maps at any relay station of the somatosensory pathway (Kaas, 1991). Functional changes were first observed in the dorsal column nuclei (Dostrovsky et al., 1976; Millar et al., 1976) and then were extensively documented in the somatosensory cortex of denervated animals (Kalaska and Pomeranz, 1979; Merzenich et al., 1983). Sensory deprivation also induces anatomical changes (i.e., neuronal damage, shrinkage of active tissue and neuropil (Kossut and Juliano, 1999; Luo and O'leary, 2005; Coleman et al., 2010). Changes are common to all species, relay stations, and sensory systems (Calford and Tweedale, 1990; Panetsos et al., 1997; Shepherd and Hardie, 2001; excellent review in Mountcastle, 2005 and Fox and Wong, 2005).

In a previous work we quantified cortical plasticity phenomena in young adult rats submitted to infraorbital nerve transection and subsequent artificial electrical neurostimulation (Herrera-Rincon et al., 2012). Sensory deprivation resulted in a clear decrease of the electrophysiological responses, metabolic activity, volume of the active neural tissue, and number of Parvalbumin- and Calbindin-positive GABA-ergic neurons, all of them consistent with previous literature (Land and Simons, 1985; Diamond et al., 1994; Glazewski et al., 1998; Huang et al., 1998; Kelly et al., 1999; Machin et al., 2004; for review, see Fox, 2002). However, all these physiological alterations, both functional and metabolic, were prevented or significantly diminished when the peripheral nerve was chronically stimulated after the transection (Herrera-Rincon et al., 2012).

Here we investigate the biocellular mechanisms and neural circuits involved in these neuroprotective processes. The best candidate was the acetylcholine (ACh) because it is heavily involved in plastic reorganization of the sensory and motor cortices and because it downregulates after peripheral deafferentation. ACh is mainly provided to the cortex by the “diffuse” corticopetal cholinergic system originated in the basal forebrain in magnocellular basal nucleus-MBN, the analogous to Meynert nucleus in primates (Mesulam et al., 1983b; Verdier and Dykes, 2001; Erzurumlu, 2003; Kamke et al., 2005; Conner et al., 2010; Saper, 2011). ACh participates in the functional reorganization of adult rat, cat and primate sensory, and motor cortices (Celesia and Jasper, 1966; Casamenti et al., 1986; Rasmusson and Dykes, 1988; Sachdev et al., 1998; Thiel et al., 2002; Golmayo et al., 2003; Weinberger, 2004). Additionally, sensory deprivation down-regulates cortical ACh through its synthesizing enzyme the choline acetyltransferase (ChAT) from the 1 days to several months after the injury (Rothe et al., 1990) indicating cholinergic circuits are affected by alterations of the sensory input (Avendano et al., 1995).

Here we address the involvement of cholinergic basalocortical system in cortical plasticity induced by peripheral input manipulations. We tested the following hypotheses: (1) cortical ACh depletion is triggered by the interruption of the sensory input; (2) the interruption of sensory input reduces ACh in MBN, consequently reducing ACh provision to the cortex; (3) The depletion of ChAT in MBN is due to the decreased activity in sensory input or to reduced trophic support. Additional questions addressed in this paper are to which extent basalocortical cholinergic system is sensory modality-specific and how deafferentation exercises a strong influence on MBN that doesn't receive direct sensory input.

Our work shows that interruption of the sensory input: reduces cortical ACh in a sensory-specific manner; acts on the cortex through MBN corticopetal neurons; is not dependent on the intensity of MBN afferent activity or on the supply of trophic factors. We discuss a possible mechanism by which peripheral deafferentation acts on MBN who doesn't receive direct sensory input.

## Materials and methods

### Experimental design, animals, and protocols

Our experimental model is the irreversible transection of the infraorbital nerve associated to a neuroprosthetic microdevice for stimulation of the proximal stump (Herrera-Rincon et al., 2012). Briefly, after complete transection of the peripheral nerve the proximal stump is inserted in a neuroprosthetic microdevice made by a silicon implant containing the electrodes that are externalized through the scalp and connected to an external stimulation device (CYGNUS-PG4000, Cygnus Tech, Delaware Water Gap, PA, USA) by means of circular connectors (Nano Circular Series; Omnetics® Connector Corporation, MN, USA) attached to the skull.

**Figure 1:**
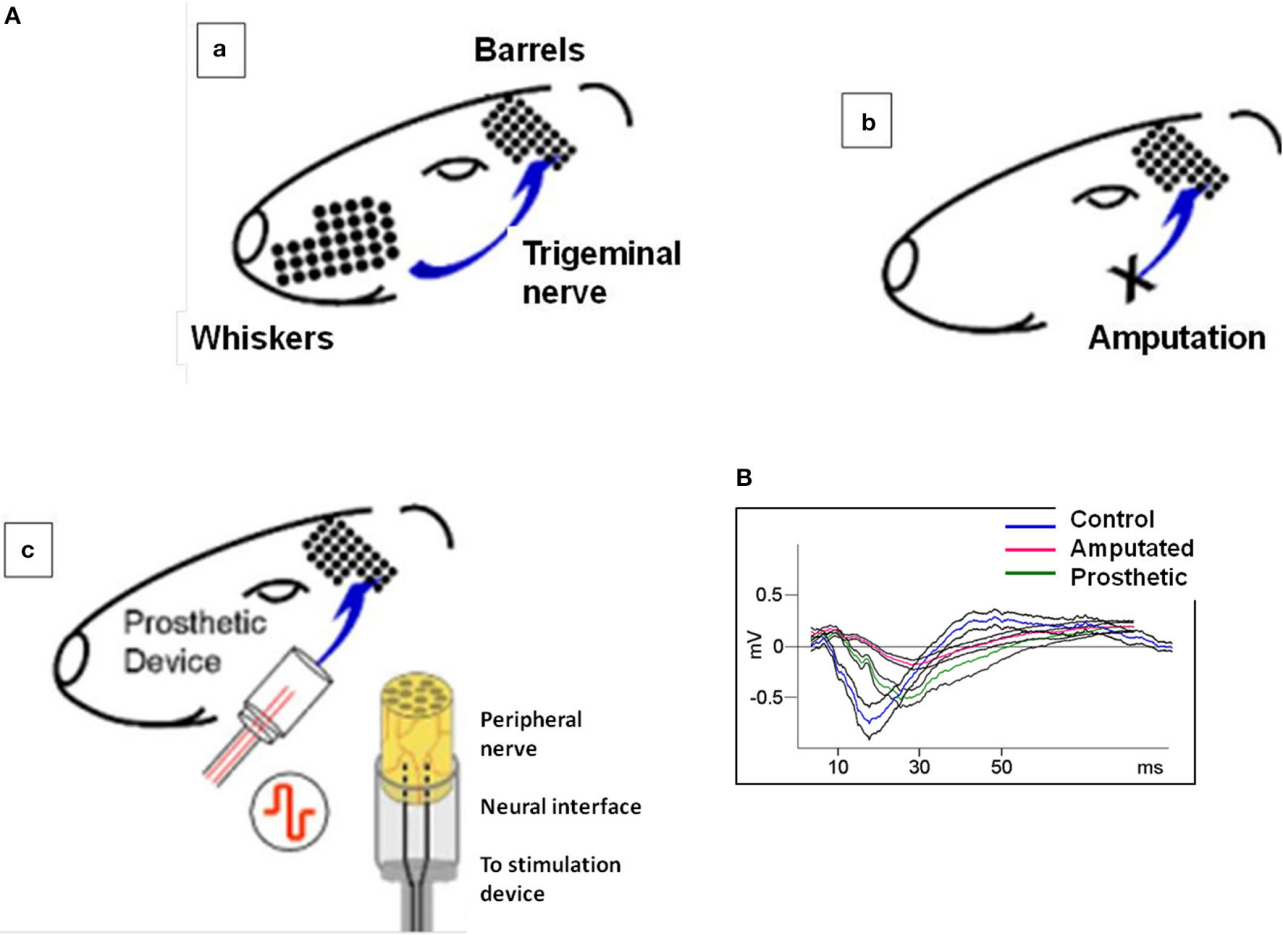
**(A)** Experimental approach: irreversible transection of the infraorbital nerve associated to a neuroprosthetic microdevice for stimulation of the proximal stump. **(a)** the rat whisker tactile system. Whiskers are arranged as a matrix in the snout of the animal. In the somatosensory cortex, there is a topographic map of the whiskers formed by large amounts of neurons receiving input from their corresponding whiskers through the trigeminal nerve and the intermediate relay stations. Pr5: nucleus principalis of the brainstem sensory trigeminal complex; VPM: ventro-posterior medial nucleus of the thalamus; BF: barrel field of the primary somatosensory cortex. **(b)** the sensory infraorbital nerve is irreversibly sectioned; the distal part of the sectioned nerve is ligated and sutured to the muscles; the proximal part of the peripheral nerve is introduced and sutured into a silicone tube containing a pair of electrodes. The sectioned peripheral nerve is regenerated into the silicone cylinder and integrated with the stimulating electrodes (magnification right). **(c)** in prosthetic animals the same procedure as in **(b)** is followed but now the implanted electrodes are connected to a stimulation device and the animal is submitted to an electrical stimulation for 4 weeks. **(B)** Typical evoked potentials recorded from the somatosensory cortex under electrical stimulation of the trigeminal nerve. Blue, red, and green lines represent the mean value of control, amputated, and prosthetic animals, respectively. Black lines represent the standard errors of 25 recordings under electrical stimulation of 200 μs-long pulses. Control evoked potentials start with a low amplitude positive component, continue with a high amplitude negative component and terminate with a lon-lasting positive wave. This behavior has not been altered in the experimental animals.

Twenty one young female adult rats were used (Wistar, 220–250 g) divided into three experimental groups: Control or C-group, with intact peripheral nerves (*n* = 5, Figure 1Aa); Amputated or A-group with animals submitted to surgical implants but without subsequent electrical stimulation (experimental controls, *n* = 9, Figure 1Ab) and Prosthetic or P-group with surgical implants and chronic electrical stimulation of the transected nerve (*n* = 7, Figure 1Ac). Stimulation started immediately after recovery from surgery and was applied for 4 weeks, 12 h per day, in square pulses of 100 μs and 3.0 V, at 20 Hz (S-group).

After 4 weeks animals were sacrificed under deep anesthesia by perfusion through the ascending aorta of 100 ml 0.9% saline, followed by 500 ml of a buffered fixative containing 4% paraformaldehyde. Brains were removed and post-fixed in the same fixative for 4–5 h. Prior to sectioning, they were cryoprotected by 2 days of immersion in buffered 30% sucrose, and cut in a cryostat (−20°C) along the horizontal plane. Consecutive series were collected for the study of different biomarkers. For the present study Nissl staining, CyO histochemistry, and ChAT immunostaining were considered.

For the anatomical tracing between deafferented somatosensory cortex and MBN, retrograde fluorescent tracer was injected in 4 additional deafferented rats (4% FG, Fluoro-Gold®, Fluorochrome, LLC, Denver, Colorado, USA). Under general anesthesia animals were placed in the stereotaxic frame, scalp was removed and an a hole was opened in the bone at the coordinates of barrel C3, approximate center of the barrel field, at 4.5 mm lateral and 2.3 mm posterior to Bregma (Chapin and Lin, 1984). Bilateral injections of the fluorescent tracer (FG, 60 nl) were made by means of a 1.0 μL blunt-point #7001 Hamilton® syringe at 1.0 mm below the dural surface. The bone was sealed with bone wax, the scalp was sutured with 4-0 nylon, and animals were allowed to survive for 7 days until sacrifice by transcardial perfusion and brain processing as above.

All surgeries were performed under general anesthesia (a mixture of ketamine 80 mg/kg i.p., and xylazine 20 mg/kg i.p.) in aseptic conditions. Animals were freely moving with free access to food and water, with 12h/12h day/night cycles. Animal handling, housing, surgery, and euthanasia were carried out in accordance with the ethical standards laid down in the 1964 Declaration of Helsinki and its later amendments, national legislation (R.D. 223/88), and EU directives on this matter (86/609/EC).

### Histochemistry and immunohistochemistry

#### Nissl staining

Sections were dipped in distilled water and stained in 0.1% cresyl violet for 15–30 min at 45°C. They were differentiated in water for 3–5 min and then dehydrated through ascending alcohols.

#### CyO histochemistry

Sections were incubated in a solution containing 0.04% cytochrome C (C7752, Sigma-Aldrich, MO, USA), 0.05% 3.3′ DAB (D8001, Sigma-Aldrich, MO, USA), and 4% sucrose in sodium phosphate buffer 0.1 M, pH 7.3, at 37°C according to the original protocol of Wong-Riley (1979).

#### ChAT immunohistochemistry

We performed indirect immunolabeling with a goat anti-ChAT monoclonal antibody (AB144P, Millipore, MA, USA) revealed by the ABC-peroxidase technique (VECTASTAIN® Elite ABC Kit, PK-6200, Vector Laboratories, USA). In most cases, the final reaction was intensified with Nickel (SK-4100, DAB Peroxidase Substrate Kit, 3,3′-diaminobenzidine, Vector Laboratories, USA). After staining, sections were mounted serially on chrome alum coated slides, dehydrated, defatted, and coverslipped.

Staining procedures were standardized and carefully performed, in order to minimize any bias or variability due to experimental procedure. In each animal the two hemispheres were simultaneously processed with the same reagents under the same conditions, so staining intensities of the two hemispheres are comparable. Moreover, to guarantee staining homogeneity animals from each group (C, A, P) were processed all together.

### Anatomical identification of relevant areas

Anatomical identification of relevant areas was carried out on Nissl- and CyO-stained sections.

*The affected primary somatosensory cortex* corresponds to the Posteromedial Barrel Subfield (PMBSF; Woolsey and Van Der Loos, 1970) clearly identifiable in CyO-labeled cortex (Land and Simons, 1985), approximately 4.6–6.6 mm laterally, −0.3 to −4.0 mm posterior to Bregma and 1.4–3.6 mm from the dural surface, corresponding to S1BF levels in Paxinos's atlas (Paxinos and Watson, 1998).

*Primary auditory cortex (Au)* was identified caudally to the PMBSF and the secondary somatosensory cortex (approximately 5.0–7.0 mm laterally, 4.0–6.4 mm posterior to Bregma, and −4.0 mm from the dorsal surface) corresponding to Au1 level in Paxinos's atlas (Paxinos and Watson, 1998).

*Primary visual cortex (Vis)* in horizontal sections was identified as the most caudal pole at dorsal levels, (approximately 2.0–4.6 mm laterally and −7.8 mm posterior to Bregma and −1.0 mm from the dural surface, corresponding to monocular (V1M) and binocular (V1B) levels in Paxinos's atlas (Paxinos and Watson, 1998).

*Anatomic delimitation of MBN* was clear in horizontal Nissl-stained and ChAT-immunoreacted sections, located caudomedial to globus pallidus (ChAT-negative staining) at amygdala and ventral pallidum level (corresponding to Ch4 group according to Mesulam nomenclature (Mesulam et al., 1983b) and reviewed in Butcher and Semba (1989).

### Cholinergic innervation and metabolic activity: optical densitometry and cell counting

#### ACh estimation procedure

ACh levels were estimated by measuring ChAT immunoexpression levels since ChAT is considered a specific biomarker for this neurotransmitter: ChAT is exclusively present into cholinergic neurons (ChAT is the limiting enzyme in ACh synthesis) and its immunoexpression is linearly correlated to ACh levels (Kuhar and Yamamura, 1976; Armstrong et al., 1983; Mesulam et al., 1983a,b; for a review see Phillis, 2005).

#### OD evaluation procedure

Cholinergic innervation levels (ChAT-immunoexpression) and regional metabolic activity (CyO-expression) in barrel, auditory, and visual cortices were estimated by the same unbiased quantitative optical densitometry (OD) procedure followed in Herrera-Rincon et al. (2012). Quantification of staining intension by means of OD is a widely used method (Masliah et al., 1990; Burke and Kenyon, 1991; Sutoo et al., 1994; Ma et al., 2001; Herrera-Rincon et al., 2012) to quantify and compare immunoexpression levels under different experimental conditions.

#### Evaluation of loss of cortically-projecting MBN neurons

**Figure 2:**
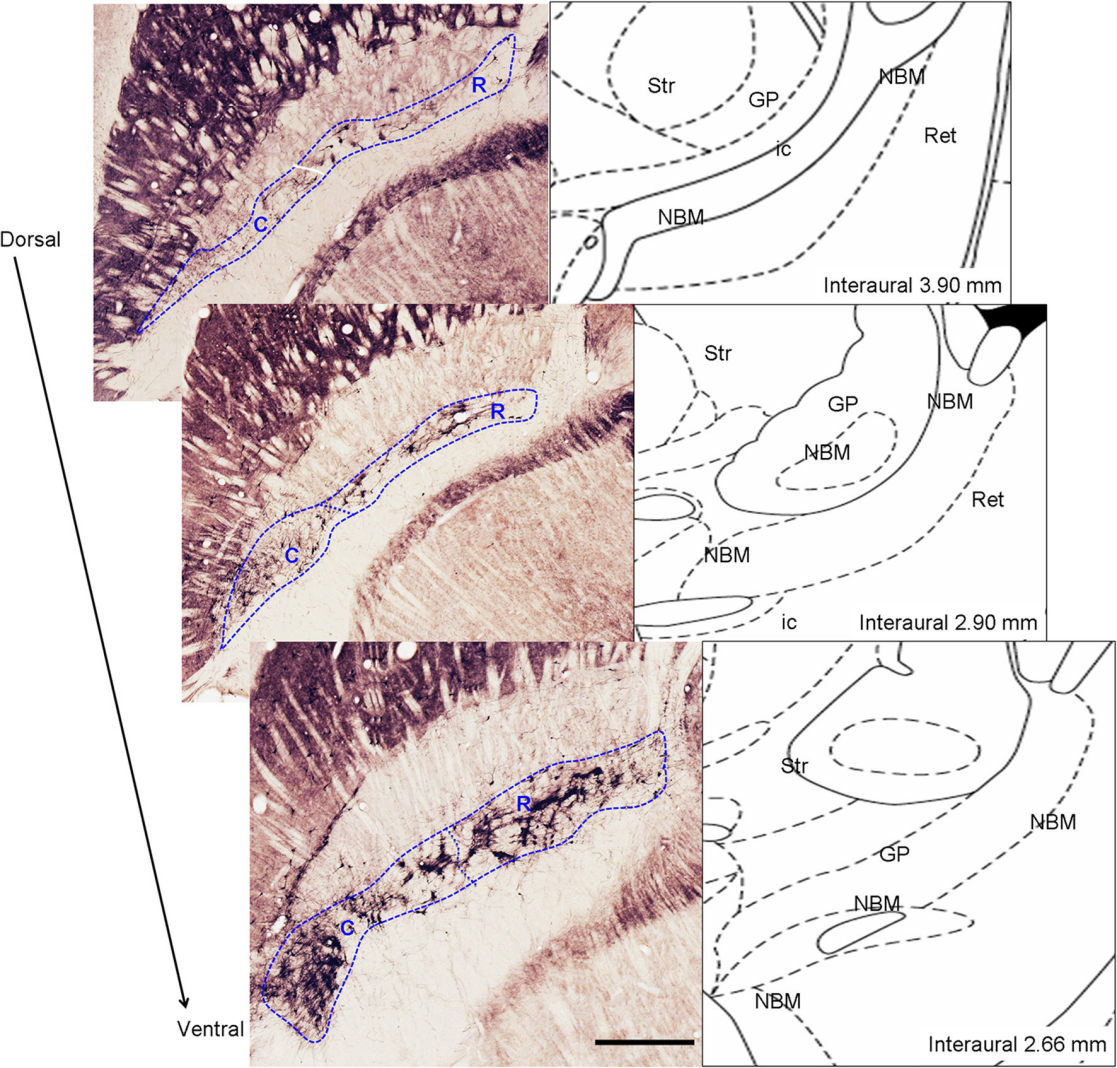
**Left:** Anatomic delimitation of magnocellular basal nucleus (MBN) and its division into rostral (R) and caudal (C) level on ChAT-immunostained horizontal sections alongside the dorso-ventral axis. **Right:** schematic representations of the sections according to the atlas of Paxinos and Watson (1998) indicating the interaural level. Str, striatum; GP, globus pallidus; MBN, magnocellular basal nucleus; ic, internal capsule; Ret, reticular thalamic nucleus. Scale bar = 500 μm.

To establish iperipheral deafferentation induces a focused loss of cortically-projecting MBN neurons and to identify the possibly affected zone we took into account the rostro-caudal topographic organization of the MBN. Rostral division is formed by neurons located dorsally and medially to the globus pallidus while caudal division is formed by the more lateral neurons, located ventro-medially with respect to the globus pallidus (Figure 2). MBN neurons projecting to medial cortical areas are located medially and rostrally while cells projecting to more lateral cortical targets occupy more lateral and caudal locations (Bigl et al., 1982; Zaborszky et al., 2013).

#### Estimation of cortical CyO and ChAT levels

In cortices OD measures were obtained from layers II/III and layer IV separately as well as the II-IV field as a whole. In detail (1) all stained tissue sections were digitized using the same bright field microscopy with planapochromatic 2.5×, 4×, 10×, 20×, and 40× dry objectives and stored to a hard disk; (2) each tissue section was sampled using a gray scale gradient of 1–256 (white to black) at a resolution of 3000 dots per inch; (3) a systematic random sampling procedure was performed both at section (approximately fraction of 1/3) and at counting frame level (approximately 1/6 from each specific cortical area); three sections were randomly selected from each brain series; in each section 5 samples of 10 × 10 pixels were obtained using a systematic random sampling procedure (4) for each sample the mean OD was calculated using ImageJ (National Institutes of Health, Maryland, USA) image processing software; (5) OD measurements were averaged for each animal; (6) in each tissue section three additional random samples were obtained from the background -unstained areas-; (7) OD correction was performed for each image by subtracting the corresponding mean background OD from the original OD value. So, for each experimental group from each animal fifteen unbiased measurements were obtained from the left and fifteen from the right hemisphere. For both CyO and ChAT staining DO values were assigned in a gray scale between 0 and 255. In order to avoid the possible inter-individual variability due to the staining procedure, for inter-group comparative analyses we employed the intra-group change percentage (contralateral vs. ipsilateral, see Statistics Section). For all measurements coefficients of error due to the sampling procedure (nugget errors, according to Cruz-Orive, 1999) ranged between 2.5 and 3.5%.

#### Counting ChAT-positive interneurons in the cortex

The majority of cortical ACh is provided by the MBN (Rye et al., 1984; Saper, 1984; Mechawar et al., 2000). According to Lehmann et al. (1980), Mechawar et al. (2000), Henny and Jones (2008), Hassani et al. (2009), and reviewed in Alitto and Dan (2012) the MBN provides the only long-range cholinergic input to the neocortex. However, MBN is not the only ACh source to the cortex since a number of cortical interneurons also provide ACh. In rodents local cholinergic interneurons in the sensory cortices (Eckenstein and Thoenen, 1983; Eckenstein and Baughman, 1984; Levey et al., 1984) account as many as 20% of ChAT-immunoreactive terminals (Eckenstein and Baughman, 1987) and they don't show profound laminar innervation patterns (Eckenstein et al., 1988). Little is known about the functions of intrinsic cholinergic neurons in the rat neocortex but they could mainly innervate blood vessels (Bayraktar et al., 1997).

#### Estimation of cortical cholinergic interneurons

Cell counts were obtained from layers II/III and layer IV separately as well as the total II-IV field as a whole, performed on horizontal ChAT-immunostained sections under 10× objective. The number of ChAT-positive cell bodies was estimated through a systematic sampling of all series of sections reacted for ChAT. From each series we considered one every 5 sections (1/5 ratio) and we counted the number of neurons in layers II-III, layer IV, and the totality of the neurons in PMBSF. Estimation of the coefficient of error for systematic random sampling was used to assess the precision of the estimates of N (Cruz-Orive, 1999). Coefficient errors ranged between 2.0 and 7.0%.

#### Estimation of loss of cholinergic corticopetal MBN neurons

In horizontal immunostained sections MBN is shown along its full rostro-caudal extension. The number of MBN ChAT-positive and FG-retrogradely labeled neurons were estimated through a systematic sampling of all such series of sections reacted for ChAT and FG, respectively. From each series we considered one every 5 sections (1/5 ratio) and we counted the totality of the neurons under a 10× objective. Estimation of ChAT-positive neurons was done by considering two anatomically different regions, rostral, and caudal (Figure 2); ChAT-immunopositive nuclei were used as counting units. For the estimation of FG-positive neurons we didn't consider rostral and caudal regions separately. Estimation of the coefficient of error for systematic random sampling was used to assess the precision of the estimates of N (Cruz-Orive, 1999). Coefficient errors ranged between 4.0 and 6.0%.

### Statistical analysis

Intra-group (inter-hemispheric) and inter-group analysis was performed for both, CyO and ChAT expression. In the first case we compared mean ipsilateral to mean contralateral OD calculating the percentage changes 100 * $\frac{OD_{CONTRALATERAL}-OD_{IPSILATERAL}}{OD_{IPSILATERAL}}$, denoted in the figures as C/I (%), for layers II/III, layer IV, and the total II-IV field for each experimental group. In the second case we compared the OD percentage changes of the experimental groups. Inter-hemispheric differences within each group were studied by paired *t*-tests. For inter-group comparisons ANOVA tests followed by *post-hoc* LSD test (when *P* < 0.05) were used. The same statistical analyses were applied in the estimation of the number of PMBSF and MBN ChAT- and FG-positive neurons. Significance level (α) was set to 0.05 in all cases. Statistical values are reported as mean ± standard error of the mean (SEM).

## Results

### Deafferentation and artificial neurostimulation effects on metabolic and cholinergic activity of the barrel somatosensory cortex

To test possible neuroprotective and plastic effects exerted by the neurostimulation on the cholinergic activity of the deafferented cortex and to compare these affects to those observed on the metabolic activity we evaluated bilateral CyO and ChAT levels and number of cholinergic interneurons in the barrel cortices of deafferented and prosthetic animals and we compared them to the cortices of the control ones. Control, amputated and prosthetic animals are denoted with C, A, and P, respectively.

To test the effectiveness of artificial stimulation and to assess the physiological conditions of the barrel cortices we recorded evoked potentials from the somatosensory cortex of C-, A-, and P- animals under electrical stimulation of 200 μs-long pulses (see Herrera-Rincon et al., 2012 for experimental details). C-animals evoked potentials started with a low amplitude positive component, continued with a high amplitude negative component and terminated with a long-lasting positive wave. A- and P-animals also displayed these three components but amplitude and duration were altered (Figure 1B).

**Figure 3:**
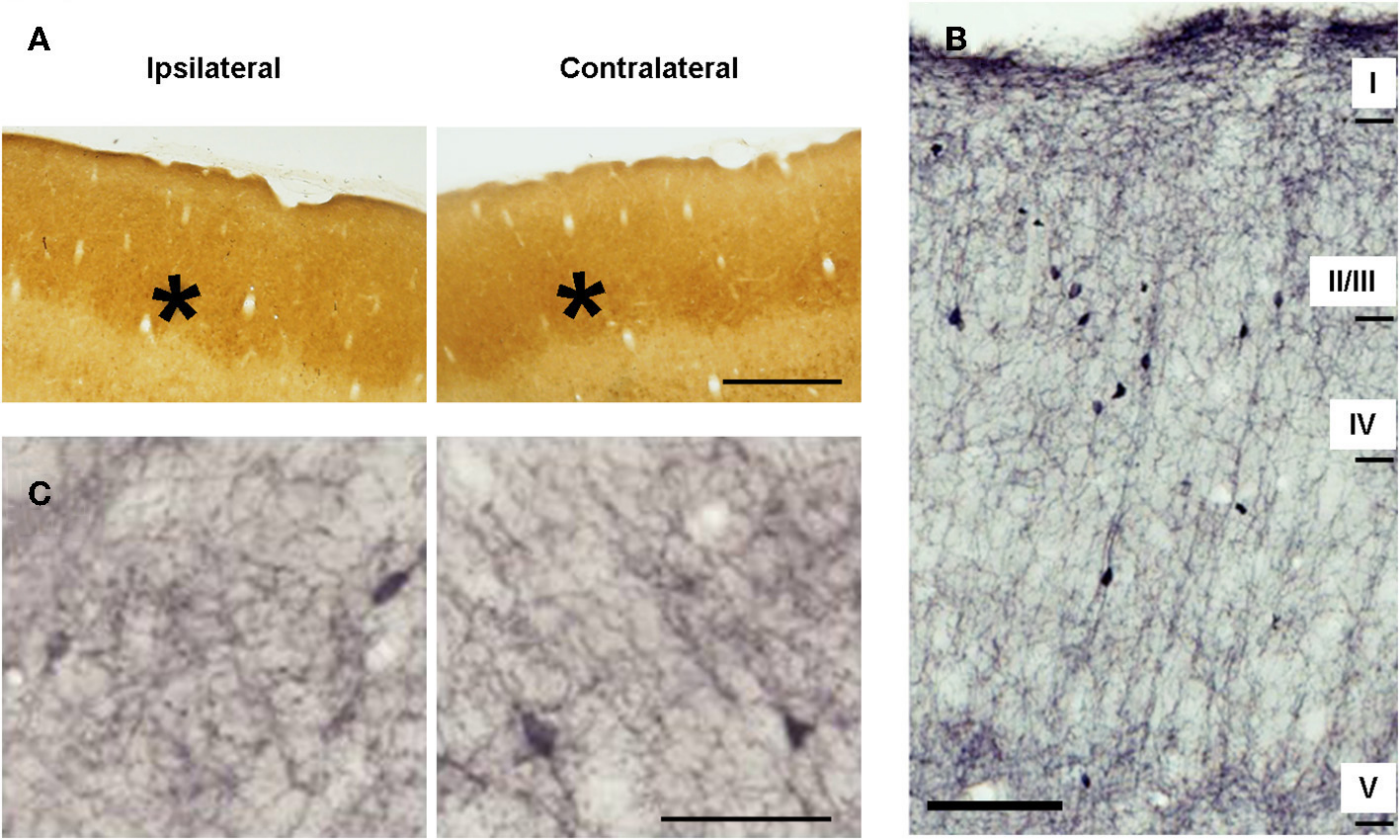
**Metabolic activity and cholinergic innervation in the barrel cortices of control animals. (A)** Microphotographs of horizontal sections of CyO-stained barrel cortices. Ipsilateral and contralateral (with respect to side of the peripheral manipulation) slides belong to sections from the same subject and were immunoreacted simultaneously. Barrels, marked with stars, are clearly visible in all sections. **(B)** Low-magnification photomicrograph showing the laminar distribution of choline acetyltransferase (ChAT)-immunopositive fibers in horizontal section from the barrel cortex. Scale bar = 100 μm. **(C)** Microphotographs of horizontal sections of ChAT-immunostained barrel cortices. Staining intensity is similar in the two cortices for both, metabolic and cholinergic activity. Panels **(A,C)** in ipsilateral hemispheres rostral is right; in contralateral hemispheres, rostral is left; lateral is top. Scale bars = 200 μm in **(A)** and 50 μm in **(C)**.

In control animals CyO staining labeled barrels in the granular layer (Figure 3A, black asterisks) related to metabolically very active thalamic-projecting zones (Wong-Riley and Welt, 1980; Land and Simons, 1985). ChAT-immunopositive cortical fibers were present in all cortical layers as a complex network of thin, homogenously distributed components. ChAT staining displayed a characteristic laminar pattern corresponding to the six cortical layers: high density of ChAT-immunostained fibers in layers I, II, upper III, and V; low density in the layers lower III and IV (Figure 3B).

**Figure 4:**
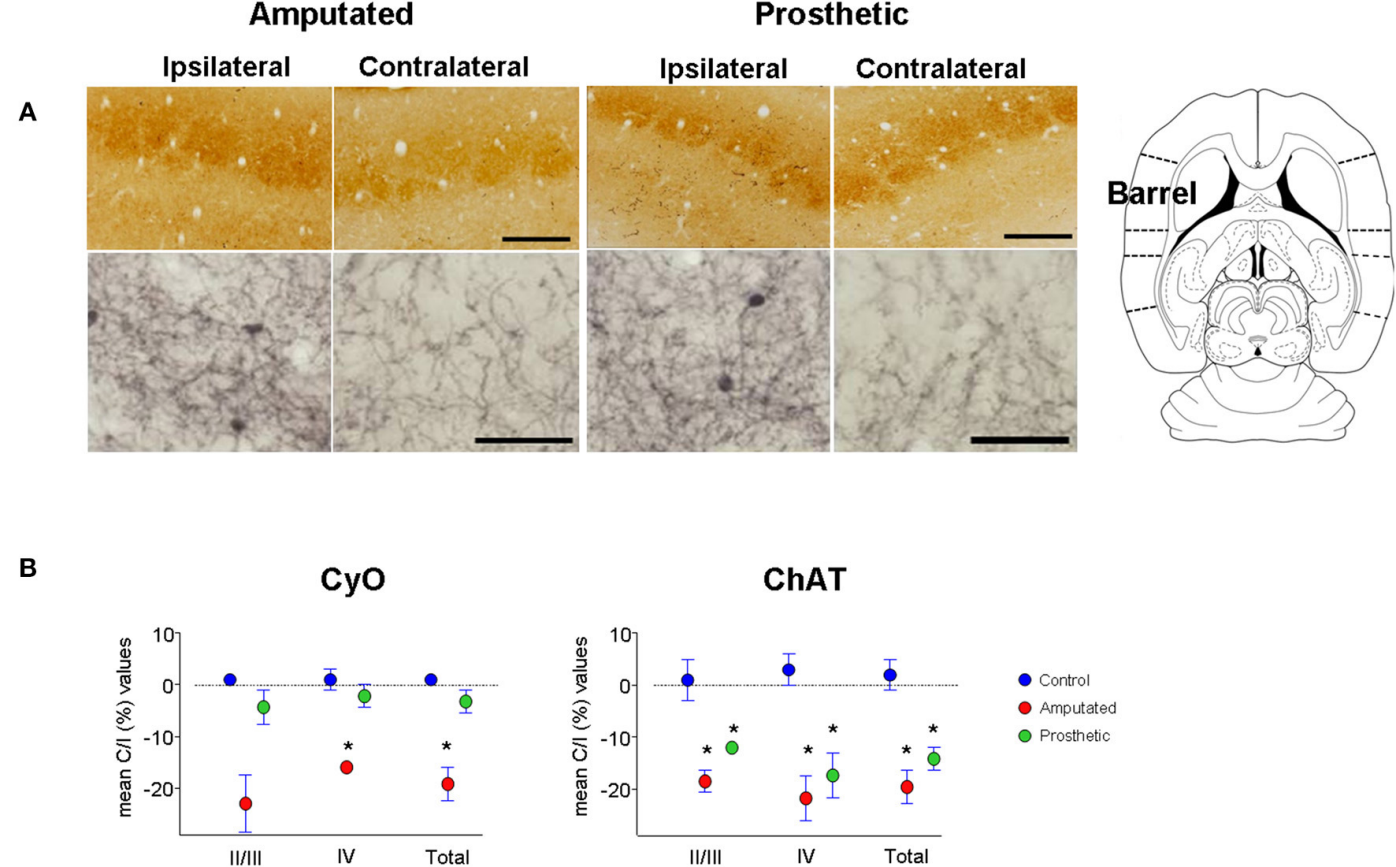
**Metabolic activity and cholinergic innervation in the barrel somatosensory cortex after amputation and after artificial stimulation of the transected infraorbital nerve. (A)** CyO-reacted and ChAT-immunostained sections are shown in upper and lower row, respectively. Ipsilateral or unaffected hemispheres are on left, contralateral or affected hemispheres are on right. Peripheral deafferentation causes a decrease in gross structure and metabolic and cholinergic states of the affected barrel cortices (Amputated-right). Artificial stimulation of the transected nerve protects the metabolic activity of the affected barrel cortices. It maintains similar CyO-levels between the two hemispheres but does not avoid the deafferentation-dependent degenerative effect on cholinergic innervation (Prosthetic-right). Ipsilateral cortices show significantly lower ChAT-staining levels than the contralateral ones. In ipsilateral hemispheres rostral is right; in contralateral hemispheres rostral is left and lateral is top. Scale bars = 200 μm in CyO; 50 μm in ChAT. **(B)** Graphic representations of mean OD percentage changes of the contralateral vs. ipsilateral hemispheres indicated as C/I (%). Metabolic activity (CyO, left) and cholinergic innervation (ChAT, right) values of the barrel cortices of control, amputated, and prosthetic animals (represented by blue, red, and green symbols, respectively) are given separately for cortical layers II/III and IV and the whole II-IV field. Values are expressed as mean ± s.e.m. Significant differences are indicated by an asterisk (ANOVA, ^*^P < 0.05).

In control animals no significant inter-hemispheric differences were observed in CyO neither in ChAT staining (Figures 3A,C and Table 1A). In A-animals sensory deprivation resulted in a dramatic decrease of both CyO and ChAT expression in the deafferented barrel cortex. Inter-hemispheric comparisons of CyO-intensity show differences of −22 ± 5%, −15 ± 1%, and −18 ± 3% for layers II/III, layer IV, and the total PMBSF, respectively and of −17 ± 2%, −20 ± 4%, and −18 ± 3% for the cholinergic neuropil (Figures 4A,B-left and Table 1B).

Table 1**(A) Statistical tables for OD CyO- reacted barrel cortex (A) and for OD ChAT- reacted barrel cortex (B)**.

**Table:** 

**(A) OD BARREL CORTEX. CyO**												
	**Intra group comparisons**											
	**Layers II/III**				**Layer IV**				**Global**			
	**Ipsi**	**Contra**	**%Δ**	***P*-value**	**Ipsi**	**Contra**	**%Δ**	***P*-value**	**Ipsi**	**Contra**	**%Δ**	***P*-value**
Control	88 ± 2	90 ± 2	−1 ± 1	0.185	106 ± 2	106 ± 2	−1 ± 2	0.799	97 ± 2	98 ± 2	1 ± 1	0.446
Amputated	80 ± 2	62 ± 1	−22 ± 5	0.077	92 ± 1	78 ± 1	−15 ± 1	0.022^*^	86 ± 1	70 ± 1	−18 ± 3	0.049^*^
Prosthetic	76 ± 1	72 ± 1	−4 ± 3	0.307	86 ± 8	84 ± 7	−2 ± 2	0.452	81 ± 1	78 ± 8	−3 ± 2	0.343

**Table:** 

	**Inter group %Δ comparisons**		
	**Layers II/III**	**Layer IV**	**Global**
Anova *P*-value	0.008^**^	0.004^**^	0.002^**^
Control vs. Amputated (*P*-value)	0.011^*^	0.002^**^	0.003^**^
Control vs. Prosthetic (*P*-value)	0.116	0.353	0.158
Amputated vs. Prosthetic (*P*-value)	0.037^*^	0.009^**^	0.016^*^

**Table:** 

**(B) OD BARREL CORTEX. ChAT**												
	**Intra group comparisons**											
	**Layers II/III**				**Layer IV**				**Global**			
	**Ipsi**	**Contra**	**%Δ**	***P*-value**	**Ipsi**	**Contra**	**%Δ**	***P*-value**	**Ipsi**	**Contra**	**%Δ**	***P*-value**
Control	48 ± 3	48 ± 1	−1 ± 4	0.875	36 ± 4	37 ± 4	3 ± 3	0.580	42 ± 4	42 ± 2	2 ± 3	0.732
Amputated	49 ± 5	41 ± 3	−17 ± 2	0.036^*^	36 ± 1	29 ± 2	−20 ± 4	0.041^*^	42 ± 2	35 ± 1	−18 ± 3	0.034^*^
Prosthetic	41 ± 4	37 ± 3	−11 ± 1	0.031^*^	37 ± 6	31 ± 6	−16 ± 4	0.033^*^	39 ± 5	34 ± 4	−13 ± 2	0.027^*^

**Table:** 

	**Inter group %Δ comparisons**		
	**Layers II/III**	**Layer IV**	**Global**
Anova *P*-value	0.008^**^	0.007^**^	0.005^**^
Control vs. Amputated (*P*-value)	0.014^*^	0.011^*^	0.011^*^
Control vs. Prosthetic (*P*-value)	0.034^*^	0.017^*^	0.016^*^
Amputated vs. Prosthetic (*P*-value)	0.095	0.525	0.255

Top: Intra group comparisons. OD values are given separately for the ipsilateral and contralateral hemispheres and layers II/III, layer IV, and global (layers II-IV). Values are presented as mean ± s.e.m. of the distribution in relative DO units. Mean percentage of interhemispheric change (%Δ) and P-value by paired t-tests are also given for both group and anatomical structure. Bottom: Inter group comparisons. One-Way ANOVA P-value is indicated on the top row. When P < 0.05, P-value for ad-hoc comparisons is indicated among Control, Amputated, and Prosthetic, two-by-two. Statistically significant differences are highlighted by

* P < 0.05,

** P < 0.01.

ns, non-significant difference.

Electrical stimulation of the transected infraorbital nerve prevented CyO-intensity from dropping in the affected hemisphere and maintained its metabolic activity at levels similar to the contralateral ones; contrary to CyO neuroprosthetic stimulation did not prevent ChAT downregulation in the deafferented cortices (Figures 4A,B-right and Tables 1A,B).

Inter-group comparisons for CyO optical density showed significant differences between C- and A-animals as well as between A- and P-animals for all three analyzed zones; no statistical differences were detected between C- and P-animals (Table 1A). Inter-group comparisons for ChAT optical density showed significant differences between C- and A-animals as well as between C- and P-animals for all three analyzed zones; No statistical differences were detected between A- and P-animals (Table 1B).

### Basal forebrain origins of cortical ChAT down-regulation

**Figure 5:**
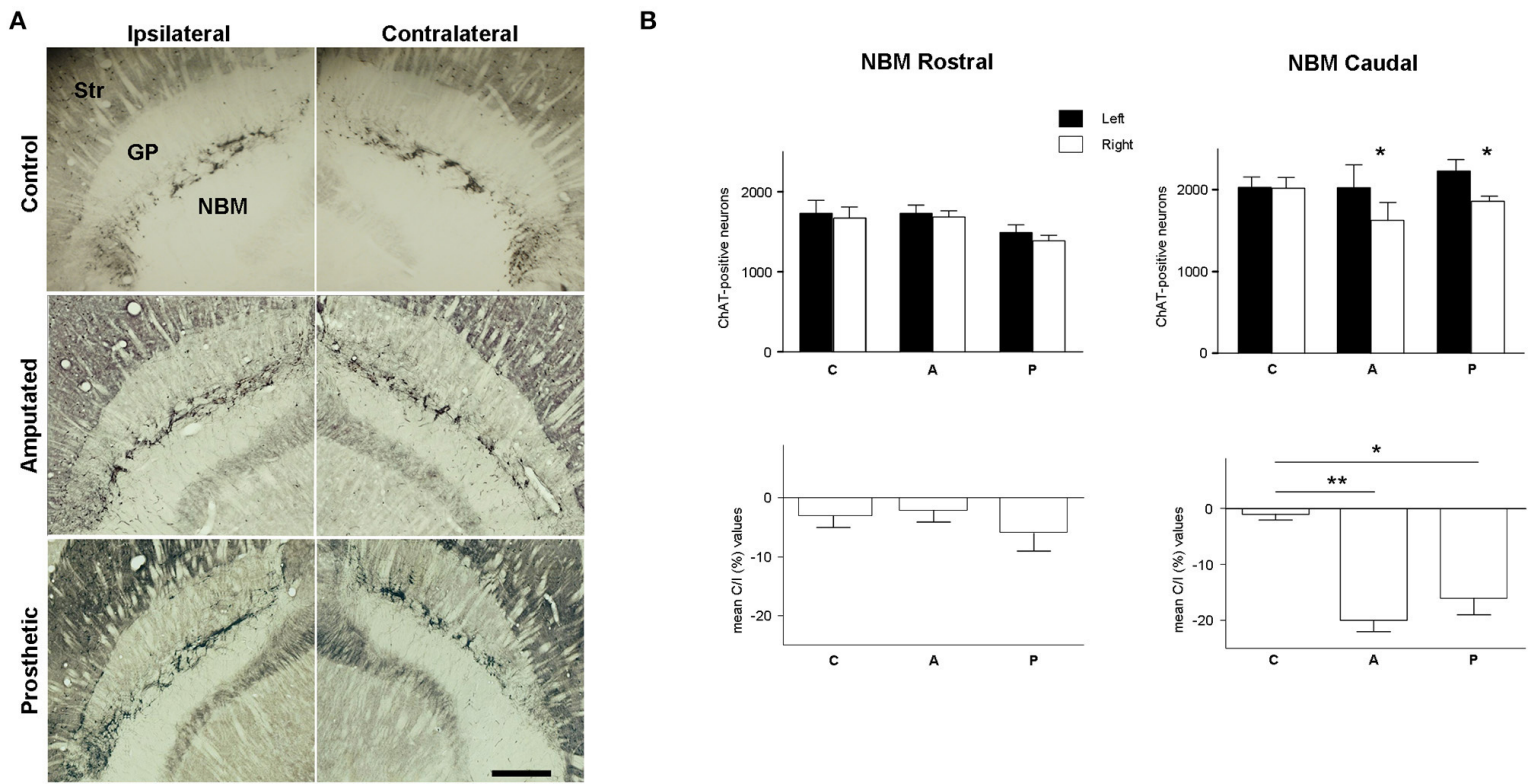
**Cholinergic neurons in magnocellular basal nucleus (MBN). (A)** Photomicrographs at low magnification of ChAT-horizontal sections, showing the rostro-caudal extension of MBN in control, amputated, and prosthetic animals. Amputated animals exhibited a significantly lower number of ChAT-positive neurons at the caudal level of the affected side. Prosthetic-animals also showed a significant loss of ChAT-positive neurons in the caudal zone of affected hemisphere. In ipsilateral hemispheres, lateral is left; in contralateral hemispheres, lateral is right; rostral is top. Str, dorsal striatum; GP, globus pallidus; Ret, reticular thalamic nucleus; VP, ventroposterior thalamic nucleus; ic, internal capsule. Scale bar = 500 μm. **(B)** Estimations of the number of cholinergic neurons in the three experimental groups for the rostral and caudal level separately. Top, inter-hemispheric comparisons (paired *t*-test, ^*^P < 0.05; ^**^P < 0.01). Bottom, inter-group comparisons (C/I (%), ANOVA ^*^P < 0.05; ^**^P < 0.01).

Next we examined the role of cortically projecting MBN neurons in such processes. The number of MBN ChAT-immunostained neurons was estimated in control, amputated, and prosthetic animals at rostral and caudal level (see Table 2, Figure 5A for microphotographs, Figure 5B for graphic representations of the results).

Table 2**MBN: estimation of ChAT-immunostained neurons in control, amputated, and prosthetic animals at rostral and caudal level of the nucleus**.

**Table:** 

**Magnocellular basal nucleus (MBN)–Number of ChAT-positive neurons**								
	**Intra group comparisons**							
	**Rostral**				**Caudal**			
	**Ipsi**	**Contra**	**% Δ**	***P*-value**	**Ipsi**	**Contra**	**% Δ**	***P*-value**
Control	1730 ± 162	1672 ± 138	−3 ± 2	0.237	2030 ± 128	2021 ± 130	−1 ± 1	0.671
Amputated	1739 ± 104	1685 ± 77	−2 ± 2	0.377	2027 ± 279	1626 ± 218	−20 ± 2	0.011^*^
Prosthetic	1490 ± 97	1388 ± 70	−6 ± 3	0.083	2229 ± 140	1859 ± 62	−16 ± 3	0.022^*^

**Table:** 

	**Inter group %Δ comparisons**	
	**Rostral**	**Caudal**
Anova *P*-value	0.444	0.001^**^
Control vs. Amputated (*P*-value)	ns	0.0^**^
Control vs. Prosthetic (*P*-value)	ns	0.010^*^
Amputated vs. Prosthetic (*P*-value)	ns	0.340

Top: Intra group comparisons. Values are presented as mean ± s.e.m. of the distribution. Mean percentage of interhemispheric change (%Δ) and P-value by paired t-tests are also given for both group and anatomical subregion. Bottom: Inter group comparisons. One-Way ANOVA P-value is indicated on the top row. When P < 0.05, P-value for ad-hoc comparisons is indicated among Control, Amputated, and Prosthetic, two-by-two. Statistically significant differences are highlighted by

* P < 0.05,

** P < 0.01.

ns, non-significant difference.

In C animals there were no significant interhemispheric differences in the counts of ChAT-positive neurons rostrally or caudally (see Table 2). Rostrally, amputation did not provoke any significant change in the number of ChAT-expressing neurons; nevertheless, caudally we faced a completely different scenario with a drastic loss of ChAT-positive neurons in the affected (contralateral) hemisphere resulting in an interhemispheric drop of 20 ± 2% (Table 2). Prosthetic animals behaved similarly to the amputated ones. No alterations were observed rostrally while the affected caudal MBN showed a drop of 16 ± 3% (see Table 2).

Rostrally inter-group comparisons did not reveal any significant difference but caudally very significant differences appear between C- and A-animals, and C- and P-animals (Table 2).

At this point we had to establish if the effect of the peripheral manipulations of the somatosensory input on the MBN is circumscribed to those cholinergic neurons that project to the somatosensory cortex. And, conversely, to determine if the damage of the cholinergic MBN neurons affects only the somatosensory cortex or if cholinergic depletion is also extended to the other sensory cortices (if it is sensory modality-specific).

**Figure 6:**
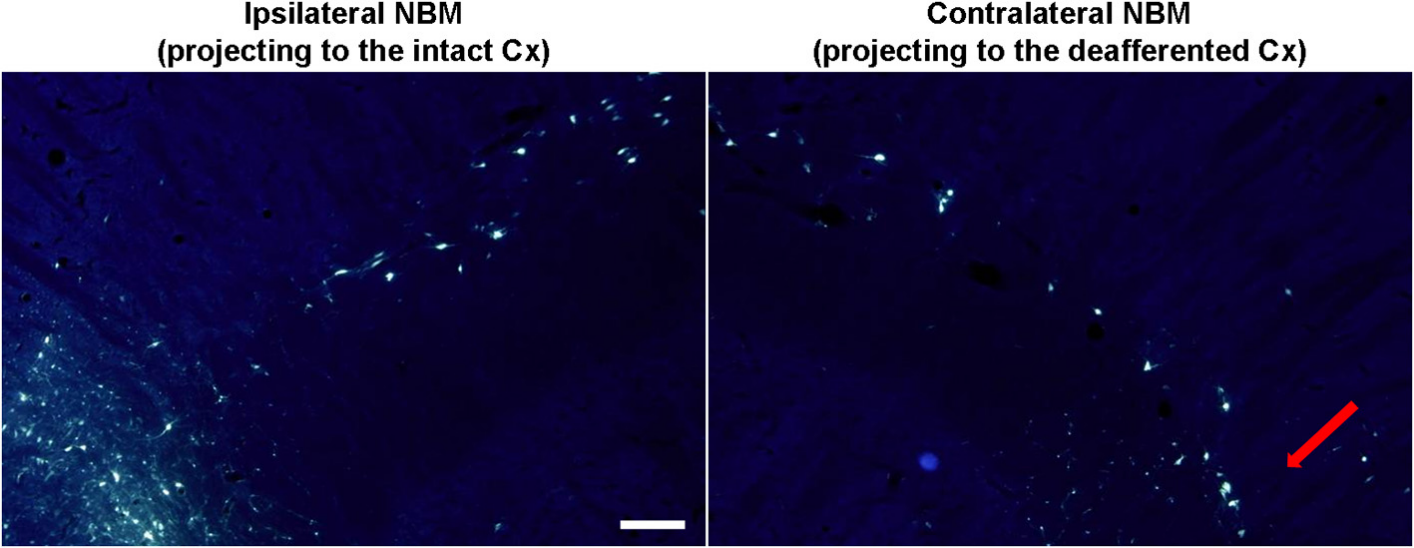
**Retrogradely labeled MBN neurons after fluoro-gold (FG) injections in the barrel cortex**. Fluorescent micrographs (UV filter) showing cell bodies in MBN that have been retrogradely labeled after bilateral-FG injection in the ipsilateral (non-affected, left) and the contralateral (deafferented, right) barrel cortex. MBN-cortical projecting neurons are diffusely scattered through MBN but they are mainly located in the caudal zone (somatosensory area, red arrow). Deafferentation results in a global neuronal damage being more intense in the specific somatosensory MBN area. In the ipsilateral hemisphere lateral is left; in the contralateral hemisphere lateral is right; rostral is top. Scale bar = 200 μm.

By means of bilateral injections of fluorescent tracers in the barrel cortices of four deafferented additional rats we retrogradely labeled MBN neurons that projected to these barrel cortices. Labeled cells were mainly localized at caudal level in both, left and right MBN (Figure 6). Qualitative morphological analysis, quantitative estimations, and inter-hemispheric comparisons revealed a significantly lower number of retrogradely labeled neurons in the MBN that projected to deafferented somatosensory cortices in all four animals. Loss of corticopetal neurons was observed alongside the whole MBN. However, absence of projecting cells was prominent in the caudal region: interhemispheric differences reached −43 ± 10% in the caudal MBN (*P* < 0.01) and −17 ± 9% in the rostral (*P* = 0.06). Caudal zone corresponded to the region with maximum drop of ChAT-positive neurons (Figure 5A).

Although intrinsic cortical cholinergic neurons account for a very low percentage of the ChAT-postive neuropil we counted the number of such acetylcholine-positive interneurons in layers II-IV in the PMBSF to estimate the contribution of such neurons in the behavior of ChAT-immunointensity in the cortical tissue. Cell counts showed similar results to the estimation of OD values. In C animals no significant mean interhemispheric differences were found for any zone (layers II/III, IV, and global PMBSF) while amputation resulted in a significant decrease in the number of ChAT-positive interneurons in the affected hemisphere in all three layers II/III (see Table 3). Cell counts in stimulated animals showed similar values and statistical results to the amputated ones (Table 3). Inter-group comparisons did not reveal significant differences among the three experimental groups for any layers (Table 3).

Table 3**Estimation of ChAT-immunostained neurons in control, amputated, and prosthetic animals in the PMBSF of the somatosensory cortex**.

**Table:** 

**Barrel Cortex (PMBSF)–Number of ChAT-positive interneurons**												
	**Intra group comparisons**											
	**Layers II/III**				**Layer IV**				**Global**			
	**Ipsi**	**Contra**	**%Δ**	***P*-value**	**Ipsi**	**Contra**	**%Δ**	***P*-value**	**Ipsi**	**Contra**	**%Δ**	***P*-value**
Control	1662 ± 30	1632 ± 48	−2 ± 5	0.766	630 ± 54	612 ± 36	−3 ± 3	0.500	2292 ± 84	2244 ± 12	−2 ± 4	0.705
Amputated	1693 ± 90	1489 ± 50	−12 ± 3	0.033^*^	620 ± 79	551 ± 57	−10 ± 3	0.081	2312 ± 161	2040 ± 104	−11 ± 3	0.033^*^
Prosthetic	1859 ± 222	1651 ± 260	−12 ± 3	0.018^*^	641 ± 91	581 ± 101	−11 ± 5	0.038^*^	2500 ± 295	2231 ± 339	−12 ± 3	0.016^*^

**Table:** 

	**Inter group %Δ comparisons**		
	**Layers II/III**	**Layer IV**	**Global**
Anova *P*-value	0.176	0.438	0.187
Control vs. Amputated (*P*-value)	ns	ns	ns
Control vs. Prosthetic (*P*-value)	ns	ns	ns
Amputated vs. Prosthetic (*P*-value)	ns	ns	ns

Top. Intra group comparisons. Values are presented as mean ± s.e.m. of the distribution. Mean percentage of interhemispheric change (%?) and P-value by paired t-tests are also given for both group and anatomical subregion. Bottom. Inter group comparisons. One-Way ANOVA P-value is indicated on the top row. When P < 0.05, P-value for ad-hoc comparisons is indicated among Control, Amputated, and Prosthetic, two-by-two. Statistically significant differences are highlighted by

* P < 0.05.

ns: non-significant difference.

### Does “somatosensory” MBN neurons downregulation affect ACh provision to other sensory cortices?

**Figure 7:**
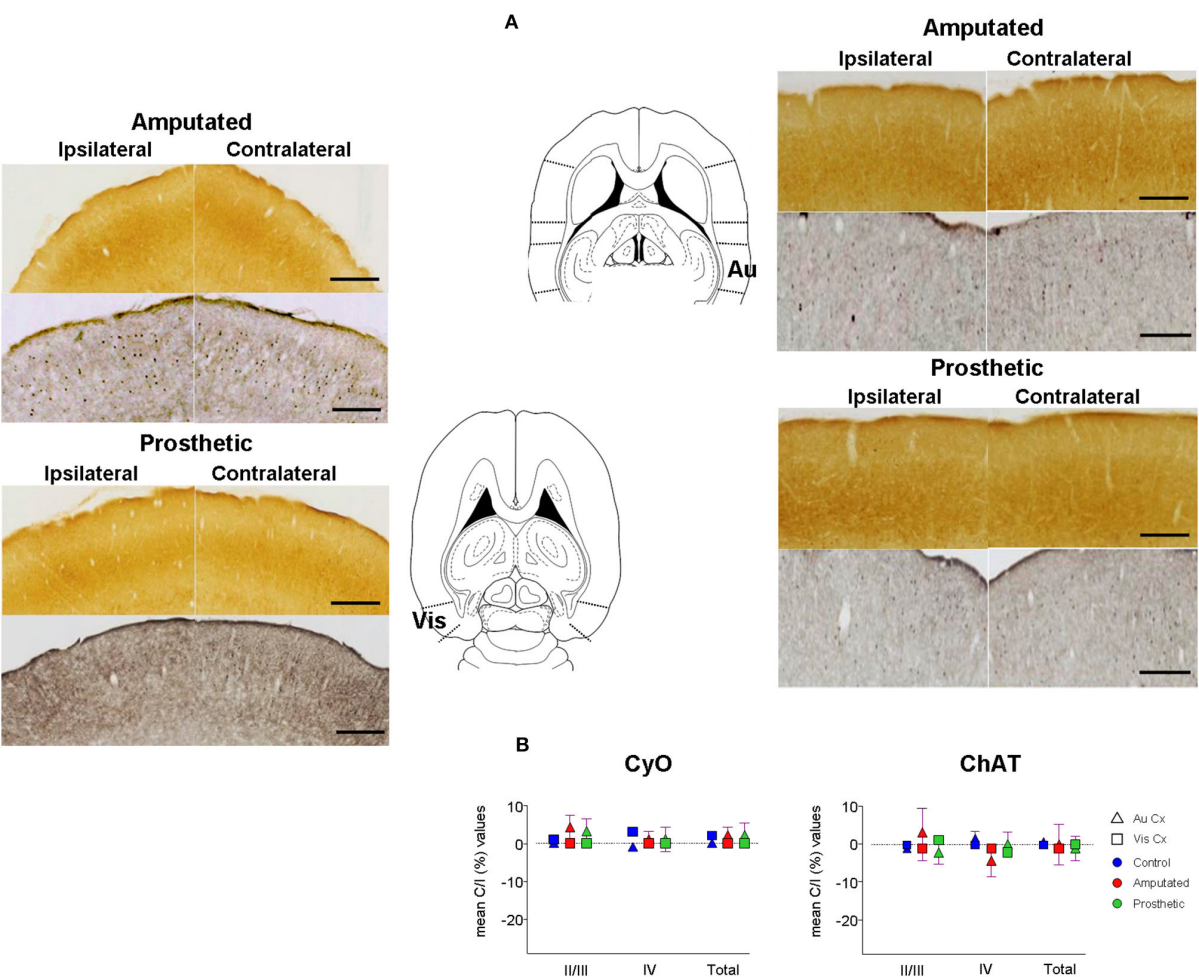
**Metabolic activity and cholinergic innervation in auditory (Au) and visual (Vis) primary sensory cortices after amputation and after artificial stimulation of the infraorbital nerve**. Deafferentation of the infraorbital nerve had no effects on the other primary sensory cortices. Also electrical stimulation had no effect. **(A)** Representative slides from the visual (left) and the auditory (right) cortex. CyO-reacted are on top and ChAT-immunostained are on bottom. Ipsilateral hemispheres are on the left, contralateral hemispheres are on the right. Drawings correspond to −3.86 and −3.10 from Bregma in horizontal diagrams according to the atlas of Paxinos and Watson (1998). In ipsilateral hemispheres rostral is right; in contralateral hemispheres, rostral is left; lateral is top. Scale bars = 200 μm for both CyO and ChAT. **(B)** Graphic representations of mean OD percentage changes of the contralateral vs. ipsilateral hemispheres indicated as C/I (%). Metabolic activity (CyO, left) and cholinergic innervation (ChAT, right) values of the visual and auditory cortices of control, amputated, and prosthetic animals (represented by blue, red, and green symbols, respectively) are given separately for cortical layers II/III and IV and the whole II-IV field. Values are expressed as mean ± s.e.m. No significant differences were found.

At this point we proposed to investigate if cortical ACh depletion due to the down-regulation of the cholinergic MBN “somatosensory” neurons is circumscribed exclusively to the somatosensory cortex or if it is also extended to other sensory cortices. To elucidate this question we performed OD analysis of CyO and ChAT stained sections of the visual and auditory cortices of C, A, and P animals (Figure 7A). Neither CyO nor ChAT showed any statistically significant intra-, and inter group differences for both sensory cortices (Figure 7B). See Tables 4A,B, for detailed CyO- and ChAT-OD values in the visual cortex, respectively, and Tables 5A,B, for CyO- and ChAT-OD values in the auditory cortex, respectively.

Table 4**Statistical tables for OD CyO- reacted visual cortex (A) and for OD ChAT- reacted visual cortex (B)**.

**Table:** 

**(A) OD VISUAL CORTEX. CyO**												
	**Intra group comparisons**											
	**Layers II/III**				**Layer IV**				**Global**			
	**Ipsi**	**Contra**	**%Δ**	***P*-value**	**Ipsi**	**Contra**	**%Δ**	***P*-value**	**Ipsi**	**Contra**	**%Δ**	***P*-value**
Control	74 ± 3	75 ± 1	0 ± 4	0.969	95 ± 1	97 ± 2	2 ± 1	0.354	85 ± 1	86 ± 1	1 ± 3	0.719
Amputated	68 ± 3	67 ± 4	−1 ± 2	0.680	86 ± 2	87 ± 1	2 ± 1	0.224	77 ± 3	77 ± 2	1 ± 0	0.081
Prosthetic	75 ± 1	74 ± 1	−1 ± 1	0.387	92 ± 1	91 ± 1	−1 ± 0	0.219	84 ± 1	83 ± 1	−1 ± 1	0.284

**Table:** 

	**Inter group %Δ comparisons**		
	**Layers II/III**	**Layer IV**	**Global**
Anova *P*-value	0.930	0.212	0.625
Control vs. Amputated (*P*-value)	ns	ns	ns
Control vs. Prosthetic (*P*-value)	ns	ns	ns
Amputated vs. Prosthetic (*P*-value)	ns	ns	ns

**Table:** 

**(B) OD VISUAL CORTEX. ChAT**												
	**Intra group comparisons**											
	**Layers II/III**				**Layer IV**				**Global**			
	**Ipsi**	**Contra**	**%Δ**	***P*-value**	**Ipsi**	**Contra**	**%Δ**	***P*-value**	**Ipsi**	**Contra**	**%Δ**	***P*-value**
Control	66 ± 6	66 ± 5	1 ± 1	0.562	50 ± 4	51 ± 6	1 ± 3	0.619	58 ± 1	59 ± 0	1 ± 2	0.583
Amputated	71 ± 3	71 ± 2	0 ± 2	0.850	48 ± 6	48 ± 7	0 ± 3	0.793	59 ± 1	59 ± 2	0 ± 2	0.966
Prosthetic	68 ± 3	70 ± 4	2 ± 3	0.522	41 ± 5	41 ± 3	−1 ± 4	0.800	55 ± 2	55 ± 2	1 ± 0	0.142

**Table:** 

	**Inter group %Δ comparisons**		
	**Layers II/III**	**Layer IV**	**Global**
Anova *P*-value	0.727	0.973	0.806
Control vs. Amputated (*P*-value)	ns	ns	ns
Control vs. Prosthetic (*P*-value)	ns	ns	ns
Amputated vs. Prosthetic (*P*-value)	ns	ns	ns

Top. Intra group comparisons. OD values are given separately for the ipsilateral and contralateral hemispheres and layers II/III, layer IV, and global (layers II-IV). Values are presented as mean ± s.e.m. of the distribution in relative DO units. Mean percentage of interhemispheric change (%Δ) and P-value by paired t-tests are also given for both group and anatomical structure. Bottom. Inter group comparisons. One-Way ANOVA P-value is indicated on the top row. When P < 0.05, P-value for ad-hoc comparisons is indicated among Control, Amputated, and Prosthetic, two-by-two. No statistically significant differences were found.

Table 5**Statistical tables for OD CyO- reacted auditory cortex (A) and for OD ChAT- reacted auditory cortex (B)**.

**Table:** 

**(A) OD AUDITORY CORTEX. CyO**												
	**Intra group comparisons**											
	**Layers II/III**				**Layer IV**				**Global**			
	**Ipsi**	**Contra**	**%Δ**	***P*-value**	**Ipsi**	**Contra**	**%Δ**	***P*-value**	**Ipsi**	**Contra**	**%Δ**	***P*-value**
Control	62 ± 2	62 ± 2	−1 ± 1	0.744	74 ± 3	72 ± 2	−2 ± 1	0.097	68 ± 1	67 ± 0	−1 ± 1	0.314
Amputated	61 ± 1	62 ± 1	3 ± 3	0.478	76 ± 3	76 ± 4	0 ± 2	0.966	69 ± 6	69 ± 6	1 ± 2	0.651
Prosthetic	67 ± 2	68 ± 1	2 ± 3	0.511	74 ± 1	74 ± 1	0 ± 3	0.910	71 ± 2	71 ± 1	1 ± 3	0.720

**Table:** 

	**Inter group %Δ comparisons**		
	**Layers II/III**	**Layer IV**	**Global**
Anova *P*-value	0.527	0.687	0.634
Control vs. Amputated (*P*-value)	ns	n	ns
Control vs. Prosthetic (*P*-value)	ns	ns	ns
Amputated vs. Prosthetic (*P*-value)	ns	ns	ns

**Table:** 

**(B) OD AUDITORY CORTEX. ChAT**												
	**Intra group comparisons**											
	**Layers II/III**				**Layer IV**				**Global**			
	**Ipsi**	**Contra**	**%Δ**	***P*-value**	**Ipsi**	**Contra**	**%Δ**	***P*-value**	**Ipsi**	**Contra**	**%Δ**	***P*-value**
Control	45 ± 6	46 ± 6	0 ± 1	0.756	39 ± 3	40 ± 4	3 ± 2	0.217	42 ± 4	43 ± 5	2 ± 1	0.332
Amputated	50 ± 0	52 ± 4	4 ± 7	0.626	41 ± 1	40 ± 2	−3 ± 4	0.637	46 ± 0	46 ± 2	1 ± 5	0.826
Prosthetic	40 ± 6	39 ± 7	−1 ± 3	0.791	35 ± 7	36 ± 8	1 ± 3	0.608	38 ± 6	38 ± 7	0 ± 3	0.831

**Table:** 

	**Inter group %Δ comparisons**		
	**Layers II/III**	**Layer IV**	**Global**
Anova *P*-value	0.667	0.513	0.872
Control vs. Amputated (*P*-value)	ns	ns	ns
Control vs. Prosthetic (*P*-value)	ns	ns	ns
Amputated vs. Prosthetic (*P*-value)	ns	ns	ns

Top. Intra group comparisons. OD values are given separately for the ipsilateral and contralateral hemispheres and layers II/III, layer IV, and global (layers II-IV). Values are presented as mean ± s.e.m. of the distribution in relative DO units. Mean percentage of interhemispheric change (%Δ) and P-value by paired t-tests are also given for both group and anatomical structure. Bottom. Inter group comparisons. One-Way ANOVA P-value is indicated on the top row. When P < 0.05, P-value for ad-hoc comparisons is indicated among Control, Amputated, and Prosthetic, two-by-two. No statistically significant differences were found.

## Discussion

### Peripheral neurostimulation of the sensory deprived somatosensory pathway does not prevent cholinergic downregulation in the deafferented cortex

Here we investigated the involvement of ACh in cortical plasticity and reorganization after peripheral deafferentation and subsequent prosthetic stimulation of the severed nerve. In a previous paper we showed that sensory neuroprosthetic input to an amputated peripheral nerve has direct and measurable neuroprotective effects on the deafferented cortices; it halts downregulations and avoids atrophies (Herrera-Rincon et al., 2012). In that study we showed that while deafferented cortices displayed a very significant reduction of electrophysiological activity, metabolism, tissue volume, and number of parvalbumin- and calbindin-expressing neurons (GABA-ergic inhibitory interneurons), all these biomarkers were maintained at normal levels after 4 weeks of artificial stimulation of the sectioned nerve. The results of the present study show that, contrary to what happens to all these cortical biomarkers, artificial stimulation is unable to prevent the degeneration of the cholinergic networks. This is not due to some error of the experimental procedure since CyO results are in line with our previous work (Herrera-Rincon et al., 2012).

Loss of ACh in the deafferented cortices is in perfect agreement with classical observations on both, immediate and persistent ACh downregulation (Rothe et al., 1990; Avendano et al., 1995). Lack of ACh recovery in stimulated animals indicates that complex plasticity and learning mechanisms are involved in cholinergic depletion and artificial stimulation cannot modulate such processes. Decrease of the cholinergic innervation alongside layers II-III and IV supports this suggestion. Indeed the cholinergic neuropil has a laminar distribution through all cortical layers and, in addition to the thalamocortical input (Metherate and Ashe, 1993), ACh synapses also contact with excitatory and inhibitory cortical interneurons and potentiate intracortically-dependent cortical responses (Sillito and Kemp, 1983; Metherate and Weinberger, 1989; Murphy and Sillito, 1991). Xiang's group (Xiang et al., 1998) suggest an inhibitory role for ACh in intracolumnar connections and a facilitatory role in intralaminar horizontal connections.

### Down-regulation of cortical acetylcholine and drop of corticopetal cholinergic neurons in the basal forebrain

Our results show that downregulation of ChAT-positive neuropil in the deafferented somatosensory cortex (Figures 4A-left,B, red symbols) is correlated to ChAT downregulation in MBN areas (Figures 5A-middle,B) which provide cholinergic innervation to the sensory-deprived cortex (Figure 6). The lower number of ChAT-positive neurons we observed in the caudal MBN (approximately 20%) of deafferented animals is compatible with classical chemoanatomical and functional data. Studies with immunotoxin IgG192–saporin in adult animals have shown that a loss of >90% of the cholinergic neurons in MBN (demonstrated by ChAT- or acetylcholinesterase (AChE)- immunohistochemistry) results in an approximately 60–80% depletion of cortical ChAT (Rossner et al., 1995; Baskerville et al., 1997), which is comparable to ACh content provided by cholinergic afferents from the MBN (Levey et al., 1983; Wahle et al., 1984), and up to >99% loss in number of AChE-positive fibers in the sensorimotor cortex (Turchi and Sarter, 1997; Conner et al., 2003), and unilateral lesions of rat MBN, either by ibotenic acid or by electrolytic lesions, decrease cortical ChAT levels by 60–50% (Wenk and Olton, 1984; Pepeu et al., 1985). Our counts of intrinsic cortical ChAT-positive neurons confirm that OD decrease is mainly due to the basalocortical input.

Local cortical effect or trophic factors like nerve growth factor and brain-derived neurotrophic factor (NGF, BDNF) have also been considered responsible for the decrease of MBN cholinergic activity (Knusel et al., 1992 and reviewed in Cuello and Bruno, 2007), probably through a retrograde effect on the expression of ChAT from the “damaged” target cortical areas (Laplante et al., 2005). Our results do not support this theory. Indeed, peripheral neurostimulation maintains the activity of the sensory deprived cortex near normal levels (Herrera-Rincon et al., 2012 and present CyO data). The normal metabolic levels and previously shown actvity levels (Herrera-Rincon et al., 2012) should have maintained NBM ChAT levels, and cholinergic actvity levels in prosthetic animals where the nerve was artificially stimulated. Such maintenance has not been observed in our experimental data (Figures 4A-right,B green symbols and Figure 5A-bottom).

### Sensory-specificity of basalocortical cholinergic system

Cortical cholinergic innervation from the MBN has been traditionally considered as a “diffuse” innervation to the whole cortical mantle and contributing to the general state awareness (Mesulam, 1995; excellent review in Sarter and Bruno, 2000). However, here we show that drop of MBN ACh due to the peripheral manipulations of the somatosensory pathway does not affect cholinergic neuropil in other primary sensory cortices like auditory or visual (Figure 7). Our findings are in agreement with more recent works showing an organized and specific pattern of cortical cholinergic innervation from the MBN depending on the connections between the targeted areas (Zaborszky et al., 2013).

Taking into account that loss of MBN neurons after the damage of the trigeminal nerve is limited to the barrel cortex-projecting region we suggest that each sensory pathway has separate access to the cholinergic MBN system. This should explain why loss of the somatosensory input or its replacement by an artificial one almost exclusively affects the “somatosensory” cholinergic circuit. In turn it should imply individual MBN neurons to be predominantly modulated by a single sensory modality and to mainly or exclusively project to the corresponding sensory areas. Our results and inferences are in agreement with (Collier and Mitchell, 1966; Neal et al., 1968; Hemsworth and Mitchell, 1969; Mullin and Phillis, 1975; Kurosawa et al., 1992) who showed that visual, auditory, and cutaneous stimulation can lead to an increase of cortical ACh in the corresponding cortical region.

How deafferentation exercises such a strong influence on the MBN which does not receive direct sensory input from the whiskers? Why artificial stimulation of the transected peripheral nerve does not normalize the down-regulated basalocortical cholinergic system? ACh down-regulation could be due to a failure in the broader neural circuit involving connections with subcortical circuits. Under normal conditions such circuits should mediate MBN activation either through intermediate nuclei (i.e., amygdala and/or hypothalamus; Irle and Markowitsch, 1986) or after being relayed by the cortex (i.e., through the posterior cortical-prefrontal-MBN circuit; Golmayo et al., 2003). The above mentioned nuclei are involved in emotional, cognitive and perception processes, learning, and memory and receive reciprocal projections from the secondary somatosensory cortex, different thalamic nuclei, etc. (Mufson et al., 1981; Cullinan and Zaborszky, 1991; Zaborszky et al., 1991; Pepeu and Blandina, 1998; Semba, 2000; Standring, 2008; Jakab et al., 2012).

### Conclusions and future perspectives

Taken together, here we have shown that cortically projecting MBN neurons provide topographically specific, functionally independent inputs that are modality specific. Our data support the existence of a modality-specific cortex-MBN-cortex circuit for cognitive information processing. Functional or context related activity may be necessary to maintain the ascending cholinergic circuit.

Due to its involvement in both cognitive processes and neuroplasticity, the cholinergic circuit represents the main target of innovative therapeutic neuroprotection-neurorehabilitation strategies. In order to be effective, neuroprosthetic-based therapeutic approaches should necessarily incorporate contextual or cognitive meaning. This might lead to the definition of new stimulation protocols based on the involvement of the cholinergic system. These stimulation protocols may be more effective for stroke or traumatic brain injury therapy and may be helpful in designing novel sensory neuroprostheses to perform in a behaviorally relevant manner in coding and transmitting sensory information to the central nervous system. Furthermore, considering the involvement of ACh in decision making and learning processes in the prefrontal cortex, new “*ad-hoc*” protocols of transcranial magnetic or current stimulation could be developed to improve learning performance or outstrip cognitive bottlenecks.

### Conflict of interest statement

The authors declare that the research was conducted in the absence of any commercial or financial relationships that could be construed as a potential conflict of interest.

## References

[B1] Alitto H. J. Dan Y. (2012). Cell-type-specific modulation of neocortical activity by basal forebrain input. Front. Syst. Neurosci. 6:79. 10.3389/fnsys.2012.00079 23316142 PMC3540901

[B2] Armstrong D. M. Saper C. B. Levey A. I. Wainer B. H. Terry R. D. (1983). Distribution of cholinergic neurons in rat brain: demonstrated by the immunocytochemical localization of choline acetyltransferase. J. Comp. Neurol. 216, 53–68. 10.1002/cne.902160106 6345598

[B3] Avendano C. Umbriaco D. Dykes R. W. Descarries L. (1995). Decrease and long-term recovery of choline acetyltransferase immunoreactivity in adult cat somatosensory cortex after peripheral nerve transections. J. Comp. Neurol. 354, 321–332. 10.1002/cne.903540302 7541804

[B4] Baskerville K. A. Schweitzer J. B. Herron P. (1997). Effects of cholinergic depletion on experience-dependent plasticity in the cortex of the rat. Neuroscience 80, 1159–1169. 10.1016/S0306-4522(97)00064-X 9284068

[B5] Bayraktar T. Staiger J. F. Acsady L. Cozzari C. Freund T. F. Zilles K. (1997). Co-localization of vasoactive intestinal polypeptide, gamma-aminobutyric acid and choline acetyltransferase in neocortical interneurons of the adult rat. Brain Res. 757, 209–217. 10.1016/S0006-8993(97)00218-7 9200749

[B6] Bigl V. Woolf N. J. Butcher L. L. (1982). Cholinergic projections from the basal forebrain to frontal, parietal, temporal, occipital, and cingulate cortices: a combined fluorescent tracer and acetylcholinesterase analysis. Brain Res. Bull. 8, 727–749. 10.1016/0361-9230(82)90101-0 6182962

[B7] Burke R. E. Kenyon N. (1991). The effect of neonatal hypoxia-ischemia on striatal cholinergic neuropil: a quantitative morphologic analysis. Exp. Neurol. 113, 63–73. 10.1016/0014-4886(91)90147-5 2044679

[B8] Butcher L. L. Semba K. (1989). Reassessing the cholinergic basal forebrain: nomenclature schemata and concepts. Trends Neurosci. 12, 483–485. 10.1016/0166-2236(89)90102-1 2480660

[B9] Calford M. B. Tweedale R. (1990). Interhemispheric transfer of plasticity in the cerebral cortex. Science 249, 805–807. 10.1126/science.2389146 2389146

[B10] Casamenti F. Deffenu G. Abbamondi A. L. Pepeu G. (1986). Changes in cortical acetylcholine output induced by modulation of the nucleus basalis. Brain Res. Bull. 16, 689–695. 10.1016/0361-9230(86)90140-1 3742251

[B11] Celesia G. G. Jasper H. H. (1966). Acetylcholine released from cerebral cortex in relation to state of activation. Neurology 16, 1053–1063. 10.1212/WNL.16.11.1053 5950916

[B12] Chapin J. K. Lin C. S. (1984). Mapping the body representation in the SI cortex of anesthetized and awake rats. J. Comp. Neurol. 229, 199–213. 10.1002/cne.902290206 6438190

[B13] Coleman J. E. Nahmani M. Gavornik J. P. Haslinger R. Heynen A. J. Erisir A.. (2010). Rapid structural remodeling of thalamocortical synapses parallels experience-dependent functional plasticity in mouse primary visual cortex. J. Neurosci. 30, 9670–9682. 10.1523/JNEUROSCI.1248-10.2010 20660250 PMC3065324

[B14] Collier B. Mitchell J. F. (1966). The central release of acetylcholine during stimulation of the visual pathway. J. Physiol. 184, 239–254. 5921540 10.1113/jphysiol.1966.sp007913 PMC1357557

[B15] Conner J. M. Culberson A. Packowski C. Chiba A. A. Tuszynski M. H. (2003). Lesions of the Basal forebrain cholinergic system impair task acquisition and abolish cortical plasticity associated with motor skill learning. Neuron 38, 819–829. 10.1016/S0896-6273(03)00288-5 12797965

[B16] Conner J. M. Kulczycki M. Tuszynski M. H. (2010). Unique contributions of distinct cholinergic projections to motor cortical plasticity and learning. Cereb. Cortex 20, 2739–2748. 10.1093/cercor/bhq022 20181623 PMC2951849

[B17] Cruz-Orive L. M. (1999). Precision of Cavalieri sections and slices with local errors. J. Microsc. 193, 182–198. 10.1046/j.1365-2818.1999.00460.x 10348655

[B18] Cuello A. C. Bruno M. A. (2007). The failure in NGF maturation and its increased degradation as the probable cause for the vulnerability of cholinergic neurons in Alzheimer's disease. Neurochem. Res. 32, 1041–1045. 10.1007/s11064-006-9270-0 17404842

[B19] Cullinan W. E. Zaborszky L. (1991). Organization of ascending hypothalamic projections to the rostral forebrain with special reference to the innervation of cholinergic projection neurons. J. Comp. Neurol. 306, 631–667. 10.1002/cne.903060408 2071698

[B20] Diamond M. E. Huang W. Ebner F. F. (1994). Laminar comparison of somatosensory cortical plasticity. Science 265, 1885–1888. 10.1126/science.8091215 8091215

[B21] Dostrovsky J. O. Millar J. Wall P. D. (1976). The immediate shift of afferent drive to dorsal column nucleus cells following deafferentation: a comparison of acute and chronic deafferentation in gracile nucleus and spinal cord. Exp. Neurol. 52, 480–495. 10.1016/0014-4886(76)90219-3 954919

[B22] Eckenstein F. Baughman R. (1987). Cholinergic innervation in cerebral cortex, in Cerebral Cortex, eds. Jones E. Peters A. (New York, NY: Plenum Press), 129–160.

[B23] Eckenstein F. Baughman R. W. (1984). Two types of cholinergic innervation in cortex, one co-localized with vasoactive intestinal polypeptide. Nature 309, 153–155. 10.1038/309153a0 6717593

[B24] Eckenstein F. P. Baughman R. W. Quinn J. (1988). An anatomical study of cholinergic innervation in rat cerebral cortex. Neuroscience 25, 457–474. 10.1016/0306-4522(88)90251-5 2456488

[B25] Eckenstein F. Thoenen H. (1983). Cholinergic neurons in the rat cerebral cortex demonstrated by immunohistochemical localization of choline acetyltransferase. Neurosci. Lett. 36, 211–215. 10.1016/0304-3940(83)90002-2 6866328

[B26] Erzurumlu R. S. (2003). Somatosensory cortical plasticity: recruiting silenced barrels by active whiskers. Exp. Neurol. 184, 565–569. 10.1016/S0014-4886(03)00396-0 14769350 PMC3671918

[B27] Fox K. (2002). Anatomical pathways and molecular mechanisms for plasticity in the barrel cortex. Neuroscience 111, 799–814. 10.1016/S0306-4522(02)00027-1 12031405

[B28] Fox K. Wong R. O. (2005). A comparison of experience-dependent plasticity in the visual and somatosensory systems. Neuron 48, 465–477. 10.1016/j.neuron.2005.10.013 16269363

[B29] Glazewski S. McKenna M. Jacquin M. Fox K. (1998). Experience-dependent depression of vibrissae responses in adolescent rat barrel cortex. Eur. J. Neurosci. 10, 2107–2116. 10.1046/j.1460-9568.1998.00222.x 9753097

[B30] Golmayo L. Nunez A. Zaborszky L. (2003). Electrophysiological evidence for the existence of a posterior cortical-prefrontal-basal forebrain circuitry in modulating sensory responses in visual and somatosensory rat cortical areas. Neuroscience 119, 597–609. 10.1016/S0306-4522(03)00031-9 12770572

[B31] Hassani O. K. Lee M. G. Henny P. Jones B. E. (2009). Discharge profiles of identified GABAergic in comparison to cholinergic and putative glutamatergic basal forebrain neurons across the sleep-wake cycle. J. Neurosci. 29, 11828–11840. 10.1523/JNEUROSCI.1259-09.2009 19776269 PMC2790860

[B32] Hemsworth B. A. Mitchell J. F. (1969). The characteristics of acetylcholine release mechanisms in the auditory cortex. Br. J. Pharmacol. 36, 161–170. 10.1111/j.1476-5381.1969.tb08313.x 5768086 PMC1703558

[B33] Henny P. Jones B. E. (2008). Projections from basal forebrain to prefrontal cortex comprise cholinergic, GABAergic and glutamatergic inputs to pyramidal cells or interneurons. Eur. J. Neurosci. 27, 654–670 10.1111/j.1460-9568.2008.06029.x 18279318 PMC2426826

[B34] Herrera-Rincon C. Torets C. Sanchez-Jimenez A. Avendano C. Panetsos F. (2012). Chronic electrical stimulation of transected peripheral nerves preserves anatomy and function in the primary somatosensory cortex. Eur. J. Neurosci. 36, 3679–3690. 10.1111/ejn.12000 23006217

[B35] Huang W. Armstrong-James M. Rema V. Diamond M. E. Ebner F. F. (1998). Contribution of supragranular layers to sensory processing and plasticity in adult rat barrel cortex. J. Neurophysiol. 80, 3261–3271. 9862920 10.1152/jn.1998.80.6.3261

[B36] Irle E. Markowitsch H. J. (1986). Afferent connections of the substantia innominata/basal nucleus of Meynert in carnivores and primates. J. Hirnforsch. 27, 343–367. 3093564

[B37] Jakab A. Molnar P. P. Bogner P. Beres M. Berenyi E. L. (2012). Connectivity-based parcellation reveals interhemispheric differences in the insula. Brain Topogr. 25, 264–271. 10.1007/s10548-011-0205-y 22002490

[B38] Kaas J. H. (1991). Plasticity of sensory and motor maps in adult mammals. Annu. Rev. Neurosci. 14, 137–167. 10.1146/annurev.ne.14.030191.001033 2031570

[B39] Kalaska J. Pomeranz B. (1979). Chronic paw denervation causes an age-dependent appearance of novel responses from forearm in “paw cortex” of kittens and adult cats. J. Neurophysiol. 42, 618–633. 422979 10.1152/jn.1979.42.2.618

[B40] Kamke M. R. Brown M. Irvine D. R. (2005). Basal forebrain cholinergic input is not essential for lesion-induced plasticity in mature auditory cortex. Neuron 48, 675–686. 10.1016/j.neuron.2005.09.014 16301182

[B41] Kelly M. K. Carvell G. E. Kodger J. M. Simons D. J. (1999). Sensory loss by selected whisker removal produces immediate disinhibition in the somatosensory cortex of behaving rats. J. Neurosci. 19, 9117–9125. 10516329 10.1523/JNEUROSCI.19-20-09117.1999 PMC6782760

[B43] Knusel B. Beck K. D. Winslow J. W. Rosenthal A. Burton L. E. Widmer H. R. (1992). Brain-derived neurotrophic factor administration protects basal forebrain cholinergic but not nigral dopaminergic neurons from degenerative changes after axotomy in the adult rat brain. J. Neurosci. 12, 4391–4402. 1432101 10.1523/JNEUROSCI.12-11-04391.1992 PMC6576000

[B44] Kossut M. Juliano S. L. (1999). Anatomical correlates of representational map reorganization induced by partial vibrissectomy in the barrel cortex of adult mice. Neuroscience 92, 807–817. 10.1016/S0306-4522(98)00722-2 10426523

[B45] Kuhar M. Yamamura H. I. (1976). Localization of cholinergic muscarinic receptors in rat brain by light microscopic radioautography. Brain Res. 110, 229–243. 10.1016/0006-8993(76)90399-1 938940

[B46] Kurosawa M. Sato A. Sato Y. (1992). Cutaneous mechanical sensory stimulation increases extracellular acetylcholine release in cerebral cortex in anesthetized rats. Neurochem. Int. 21, 423–427. 10.1016/0197-0186(92)90194-V 1303167

[B47] Land P. W. Simons D. J. (1985). Metabolic and structural correlates of the vibrissae representation in the thalamus of the adult rat. Neurosci. Lett. 60, 319–324. 10.1016/0304-3940(85)90597-X 2999649

[B48] Laplante F. Morin Y. Quirion R. Vaucher E. (2005). Acetylcholine release is elicited in the visual cortex, but not in the prefrontal cortex, by patterned visual stimulation: a dual *in vivo* microdialysis study with functional correlates in the rat brain. Neuroscience 132, 501–510 10.1016/j.neuroscience.2004.11.059 15802200

[B49] Lehmann J. Nagy J. I. Atmadia S. Fibiger H. C. (1980). The magnocellular basal nucleus: the origin of a cholinergic projection to the neocortex of the rat. Neuroscience 5, 1161–1174. 10.1016/0306-4522(80)90195-5 7402465

[B51] Levey A. I. Wainer B. H. Mufson E. J. Mesulam M. M. (1983). Co-localization of acetylcholinesterase and choline acetyltransferase in the rat cerebrum. Neuroscience 9, 9–22. 10.1016/0306-4522(83)90042-8 6348584

[B52] Levey A. I. Wainer B. H. Rye D. B. Mufson E. J. Mesulam M. M. (1984). Choline acetyltransferase-immunoreactive neurons intrinsic to rodent cortex and distinction from acetylcholinesterase-positive neurons. Neuroscience 13, 341–353. 10.1016/0306-4522(84)90234-3 6514183

[B53] Luo L. O'leary D. D. (2005). Axon retraction and degeneration in development and disease. Annu. Rev. Neurosci. 28, 127–156. 10.1146/annurev.neuro.28.061604.135632 16022592

[B54] Ma T. Cai Z. Wellman S. E. Ho I. K. (2001). A quantitative histochemistry technique for measuring regional distribution of acetylcholinesterase in the brain using digital scanning densitometry. Anal. Biochem. 296, 18–28. 10.1006/abio.2001.5208 11520028

[B55] Machin R. Blasco B. Bjugn R. Avendano C. (2004). The size of the whisker barrel field in adult rats: minimal nondirectional asymmetry and limited modifiability by chronic changes of the sensory input. Brain Res. 1025, 130–138. 10.1016/j.brainres.2004.07.077 15464753

[B56] Masliah E. Terry R. D. Alford M. Deteresa R. (1990). Quantitative immunohistochemistry of synaptophysin in human neocortex: an alternative method to estimate density of presynaptic terminals in paraffin sections. J. Histochem. Cytochem. 38, 837–844. 10.1177/38.6.2110586 2110586

[B57] Mechawar N. Cozzari C. Descarries L. (2000). Cholinergic innervation in adult rat cerebral cortex: a quantitative immunocytochemical description. J. Comp. Neurol. 428, 305–318. 10.1002/1096-9861(20001211)428:2<305::AID-CNE9>3.0.CO;2-Y 11064369

[B58] Merzenich M. M. Kaas J. H. Wall J. Nelson R. J. Sur M. Felleman D. (1983). Topographic reorganization of somatosensory cortical areas 3b and 1 in adult monkeys following restricted deafferentation. Neuroscience 8, 33–55. 10.1016/0306-4522(83)90024-6 6835522

[B59] Mesulam M. M. (1995). Cholinergic pathways and the ascending reticular activating system of the human brain. Ann. N.Y. Acad. Sci. 757, 169–179. 10.1111/j.1749-6632.1995.tb17472.x 7611672

[B60] Mesulam M. M. Mufson E. J. Levey A. I. Wainer B. H. (1983a). Cholinergic innervation of cortex by the basal forebrain: cytochemistry and cortical connections of the septal area, diagonal band nuclei, nucleus basalis (substantia innominata), and hypothalamus in the rhesus monkey. J. Comp. Neurol. 214, 170–197. 10.1002/cne.902140206 6841683

[B61] Mesulam M. M. Mufson E. J. Wainer B. H. Levey A. I. (1983b). Central cholinergic pathways in the rat: an overview based on an alternative nomenclature (Ch1-Ch6). Neuroscience 10, 1185–1201. 10.1016/0306-4522(83)90108-2 6320048

[B62] Metherate R. Ashe J. H. (1993). Nucleus basalis stimulation facilitates thalamocortical synaptic transmission in the rat auditory cortex. Synapse 14, 132–143. 10.1002/syn.890140206 8392756

[B63] Metherate R. Weinberger N. M. (1989). Acetylcholine produces stimulus-specific receptive field alterations in cat auditory cortex. Brain Res. 480, 372–377. 10.1016/0006-8993(89)90210-2 2713663

[B64] Millar J. Basbaum A. I. Wall P. D. (1976). Restructuring of the somatotopic map and appearance of abnormal neuronal activity in the gracile nucleus after partial deafferentation. Exp. Neurol. 50, 658–672. 10.1016/0014-4886(76)90035-2 1253869

[B65] Mountcastle V. (2005). The Sensory Hand: Neural Mechanisms of Somatic Sensation. Cambridge, MA: Harvard University Press.

[B66] Mufson E. J. Mesulam M. M. Pandya D. N. (1981). Insular interconnections with the amygdala in the rhesus monkey. Neuroscience 6, 1231–1248. 10.1016/0306-4522(81)90184-6 6167896

[B67] Mullin W. J. Phillis J. W. (1975). The effects of graded forelimb afferent volleys on acetylcholine release from cat sensorimotor cortex. J. Physiol. 244, 741–756. 1133777 10.1113/jphysiol.1975.sp010823 PMC1330833

[B68] Murphy P. C. Sillito A. M. (1991). Cholinergic enhancement of direction selectivity in the visual cortex of the cat. Neuroscience 40, 13–20. 10.1016/0306-4522(91)90170-S 2052147

[B69] Neal M. J. Hemsworth B. A. Mitchell J. F. (1968). The excitation of central cholinergic mechanisms by stimulation of the auditory pathway. Life Sci. 7, 757–763 10.1016/0024-3205(68)90132-X

[B70] Panetsos F. Nunez A. Avendano C. (1997). Electrophysiological effects of temporary deafferentation on two characterized cell types in the nucleus gracilis of the rat. Eur. J. Neurosci. 9, 563–572. 10.1111/j.1460-9568.1997.tb01633.x 9104598

[B71] Paxinos G. Watson C. (1998). The Rat Brain in Stereotaxic Coordinates. San Diego, CA: Academic Press.

[B72] Pepeu G. Blandina P. (1998). The acetylcholine, GABA, glutamate triangle in the rat forebrain. J. Physiol. Paris 92, 351–355. 10.1016/S0928-4257(99)80004-7 9789836

[B73] Pepeu G. Casamenti F. Bracco L. Ladinsky H. Consolo S. (1985). Lesions of the nucleus basalis in the rat: functional changes, in Senile Dementia of the Alzheimer Type, eds Traber J. Gispen W. (Berlin; Heidelberg: Springer), 305–315.

[B74] Phillis J. W. (2005). Acetylcholine release from the central nervous system: a 50-year retrospective. Crit. Rev. Neurobiol. 17, 161–217. 10.1615/CritRevNeurobiol.v17.i3-4.30 17341198

[B75] Rasmusson D. D. Dykes R. W. (1988). Long-term enhancement of evoked potentials in cat somatosensory cortex produced by co-activation of the basal forebrain and cutaneous receptors. Exp. Brain Res. 70, 276–286. 10.1007/BF00248353 3384031

[B76] Rossner S. Schliebs R. Bigl V. (1995). 192IgG-saporin-induced immunotoxic lesions of cholinergic basal forebrain system differentially affect glutamatergic and GABAergic markers in cortical rat brain regions. Brain Res. 696, 165–176. 10.1016/0006-8993(95)00844-G 8574666

[B77] Rothe T. Hanisch U. K. Krohn K. Schliebs R. Hartig W. Webster H. H. (1990). Changes in choline acetyltransferase activity and high-affinity choline uptake, but not in acetylcholinesterase activity and muscarinic cholinergic receptors, in rat somatosensory cortex after sciatic nerve injury. Somatosens. Mot. Res. 7, 435–446 10.3109/08990229009144718 1963253

[B78] Rye D. B. Wainer B. H. Mesulam M. M. Mufson E. J. Saper C. B. (1984). Cortical projections arising from the basal forebrain: a study of cholinergic and noncholinergic components employing combined retrograde tracing and immunohistochemical localization of choline acetyltransferase. Neuroscience 13, 627–643. 10.1016/0306-4522(84)90083-6 6527769

[B79] Sachdev R. N. Lu S. M. Wiley R. G. Ebner F. F. (1998). Role of the basal forebrain cholinergic projection in somatosensory cortical plasticity. J. Neurophysiol. 79, 3216–3228. 9636120 10.1152/jn.1998.79.6.3216

[B80] Saper C. B. (1984). Organization of cerebral cortical afferent systems in the rat. II. Magnocellular basal nucleus. J. Comp. Neurol. 222, 313–342. 10.1002/cne.902220302 6699210

[B81] Saper C. B. (2011). Diffuse cortical projection systems: anatomical organization and role in cortical function, in Comprehensive Physiology 2011, Supplement 5: Handbook of Physiology, The Nervous System, Higher Functions of the Brain, ed Pollock D. M. (Georgia Regents University), 169–210 10.1002/cphy.cp010506

[B82] Sarter M. Bruno J. P. (2000). Cortical cholinergic inputs mediating arousal, attentional processing and dreaming: differential afferent regulation of the basal forebrain by telencephalic and brainstem afferents. Neuroscience 95, 933–952. 10.1016/S0306-4522(99)00487-X 10682701

[B83] Semba K. (2000). Multiple output pathways of the basal forebrain: organization, chemical heterogeneity, and roles in vigilance. Behav. Brain Res. 115, 117–141. 10.1016/S0166-4328(00)00254-0 11000416

[B84] Shepherd R. K. Hardie N. A. (2001). Deafness-induced changes in the auditory pathway: implications for cochlear implants. Audiol. Neurootol. 6, 305–318. 10.1159/000046843 11847461

[B85] Sillito A. M. Kemp J. A. (1983). Cholinergic modulation of the functional organization of the cat visual cortex. Brain Res. 289, 143–155. 10.1016/0006-8993(83)90015-X 6661640

[B86] Standring S. (2008). Gray's Anatomy: the Anatomical Basis of Clinical Practice. Edinburgh: Churchill Livingstone/Elsevier.

[B88] Sutoo D. Akiyama K. Yabe K. Kohno K. (1994). Quantitative analysis of immunohistochemical distributions of cholinergic and catecholaminergic systems in the human brain. Neuroscience 58, 227–234. 10.1016/0306-4522(94)90170-8 7909146

[B89] Thiel C. M. Friston K. J. Dolan R. J. (2002). Cholinergic modulation of experience-dependent plasticity in human auditory cortex. Neuron 35, 567–574. 10.1016/S0896-6273(02)00801-2 12165477

[B90] Turchi J. Sarter M. (1997). Cortical acetylcholine and processing capacity: effects of cortical cholinergic deafferentation on crossmodal divided attention in rats. Brain Res. Cogn. Brain Res. 6, 147–158. 10.1016/S0926-6410(97)00027-X 9450608

[B91] Verdier D. Dykes R. W. (2001). Long-term cholinergic enhancement of evoked potentials in rat hindlimb somatosensory cortex displays characteristics of long-term potentiation. Exp. Brain Res. 137, 71–82. 10.1007/s002210000646 11310174

[B92] Wahle P. Sanides-Buchholtz C. Eckenstein F. Albus K. (1984). Concurrent visualization of choline acetyltransferase-like immunoreactivity and retrograde transport of neocortically injected markers in basal forebrain neurons of cat and rat. Neurosci. Lett. 44, 223–228. 10.1016/0304-3940(84)90026-0 6374512

[B93] Weinberger N. M. (2004). Specific long-term memory traces in primary auditory cortex. Nat. Rev. Neurosci. 5, 279–290. 10.1038/nrn1366 15034553 PMC3590000

[B94] Wenk G. L. Olton D. S. (1984). Recovery of neocortical choline acetyltransferase activity following ibotenic acid injection into the nucleus basalis of Meynert in rats. Brain Res. 293, 184–186. 10.1016/0006-8993(84)91468-9 6704718

[B95] Wong-Riley M. (1979). Changes in the visual system of monocularly sutured or enucleated cats demonstrable with cytochrome oxidase histochemistry. Brain Res. 171, 11–28. 10.1016/0006-8993(79)90728-5 223730

[B96] Wong-Riley M. T. Welt C. (1980). Histochemical changes in cytochrome oxidase of cortical barrels after vibrissal removal in neonatal and adult mice. Proc. Natl. Acad. Sci. U.S.A. 77, 2333–2337. 10.1073/pnas.77.4.2333 6246540 PMC348709

[B97] Woolsey T. A. Van Der Loos H. (1970). The structural organization of layer IV in the somatosensory region (SI) of mouse cerebral cortex. The description of a cortical field composed of discrete cytoarchitectonic units. Brain Res. 17, 205–242. 10.1016/0006-8993(70)90079-X 4904874

[B98] Xiang Z. Huguenard J. R. Prince D. A. (1998). Cholinergic switching within neocortical inhibitory networks. Science 281, 985–988. 10.1126/science.281.5379.985 9703513

[B99] Zaborszky L. Csordas A. Mosca K. Kim J. Gielow M. R. Vadasz C.. (2013). Neurons in the basal forebrain project to the cortex in a complex topographic organization that reflects corticocortical connectivity patterns: an experimental study based on retrograde tracing and 3D reconstruction. Cereb. Cortex. [Epub ahead of print]. 10.1093/cercor/bht210. 23964066 PMC4259277

[B100] Zaborszky L. Cullinan W. E. Braun A. (1991). Afferents to basal forebrain cholinergic projection neurons: an update. Adv. Exp. Med. Biol. 295, 43–100. 10.1007/978-1-4757-0145-6_2 1776580

